# Trace Cd^2+^ Ions Detection on the Flower-Like Ag@CuO Substrate

**DOI:** 10.3390/nano10091664

**Published:** 2020-08-25

**Authors:** Mingming Cheng, Chenyan Li, Weijun Li, Yingkai Liu

**Affiliations:** 1 Yunnan Key Laboratory of Opto-electronic Information Technology, Yunnan Normal University, Kunming 650500, China; Chengminng0526@126.com (M.C.); Lichenyan12@126.com (C.L.); Liweijun0009@126.com (W.L.); 2 Institute of Physics and Electronic Information, Yunnan Normal University, Kunming 650500, China

**Keywords:** Ag@CuO FM, surface enhanced raman scattering, rhodamine 6G, cadmium ion

## Abstract

CuO flower-like material (FM) was prepared via the facile hydrothermal method, and Ag nanoparticles were deposited on the CuO FM to obtain Ag@CuO composite. Rhodamine 6G (R6G) was used as the probe molecule on Ag@CuO FM substrate to study surface enhanced Raman scattering (SERS). It is discovered that it exhibited an excellent SERS performance with limit of detection of 3.58 × 10^−16^ M and enhancement factor (EF) of 3.99 × 10^10^. More importantly, we used it as a SERS substrate to detect cadmium ions and found that its limit of detection (LOD) reaches up to 2.6 × 10^−8^ M, which is lower than the highest allowable Cd^2+^ concentration in drinking water set by the World Health Organization (WHO) and Environmental Protection Agency (EPA). Therefore, the proposed composite can be applicable to the detection of Cd^2+^ in drinking water and in soil.

## 1. Introduction

Cadmium is deemed to be an extremely toxic heavy metal, which leads to severe nephropathies [1]. In general, cadmium is omnipresent and has been widely exploited in mining, plastic, erosion, volcanic activity, nuclear power plants, and so on [2]. These applications significantly raise the underlying pollution to the environment [3]. Now, the highest allowable concentration of Cd^2+^ in drinking water is 3 μg·L^−1^ and 5 μg·L^−1^ specified by the World Health Organization (WHO) and Environmental Protection Agency (EPA), respectively [4,5]. Based on people’s concerns, various methods have been developed to achieve trace detection for the determination of Cd^2+^ ions, for example, electrochemical methods [6], atomic absorption/emission spectrometry [7,8], inductively coupled plasma mass spectrometry (ICP–MS) [9], and fluorescence spectroscopy [3]. However, their actual applications have been limited for the reason that they are cumbersome in specimen preparation and unsuitable for on-site measurement. Therefore, in order to meet the urgent needs of society, we need to study a simple and effective way to detect Cd^2+^.

It is well known that Raman spectroscopy was discovered by Raman and Krishnan [10] in 1928 and has been used as an analysis tool since its discovery. However, there are some limitations for the conventional Raman in tracing analysis. For example, since the Raman scattering cross section is much smaller than fluorescence, its signal is very weak [11]. Fortunately, Fleischmann et al. [12] and Jeanmaire et al. [13] discovered that when the analyte is disposed at or near the surface roughness of the noble metal, the Raman signal can be promoted to a million times stronger than that of a conventional Raman signal [13]. Afterwards, noble and transition metals such as silver, gold, copper, and palladium [14] have been greatly improved. However, it is required that substrate possesses not only high sensitivity and stability, but also low cost in practice application. Consequently, precious metals are not the best choice for surface enhanced Raman scattering (SERS) substrate. It was documented that semiconductor nanomaterials (SNs) such as ZnO [15], Cu_2_O [16], and CuO [17,18,19] also produce weak SERS signals. The composite structure of precious metals and semiconductor nanomaterials has a better effect on SERS [20] because it has not only the electromagnetic enhancement effect between precious metal nanoparticles, but also the chemical enhancement one produced by SNs. The two aspects make up for each other’s shortcomings and merge their respective merits.

Recently, it was demonstrated that it is an effective way to integrate the noble nanoparticles (NPs) with the SNs as to enhance SERS performance [21]. For instance, the Ag@CuO superhydrophobic nanomaterial-based SERS substrate [17] shows enhanced Raman spectrum with an enhancement factor (EF) of 2.0 × 10^7^ and limit of detection (LOD) of 10^−10^ M for rhodamine 6G (R6G). Au NPs/ carbon nanosheets [22] could generate strong Raman signal (EF ~1.2 × 10^6^) with well uniformity and repeatability for 4-34 aminothiophenol. The Ag@ZnO hierarchical nanorod arrays [15] possessed large EF up to 4.2 × 10^7^ for R6G. Therefore, it is of vital importance to prepare optimal hybrid semiconductors for detecting chemical analytes [18], deoxyribonucleic acid (DNA) analysis [23], environmental contaminants [24], and so on.

Here, we prepared Ag@CuO flower-like material (FM) and investigated its SERS property by using R6G as probe molecules. It was revealed that the substrate exhibited limit of detection (LOD) of 3.58 × 10^−16^ M and EF of 3.99 × 10^10^ for R6G. Due to its excellent SERS performance, we hope that it can be extended to detect heavy metal ions as to solve environmental pollution. Therefore, the Ag@CuO FM was used as a substrate and Pb^2+^, Cd^2+^, and Hg^2+^ ions as probe molecules for SERS detection. It was found that this substrate has better selectivity to Cd^2+^ ions with EF of 6.9 × 10^3^ and LOD of 2.6 × 10^−8^ M. This study opens up a new possibility for rapid, simple, and reliable diagnosis of Cd^2+^ in the environment.

## 2. Materials and Methods

### 2.1. Reagents 

Copper chloride (CuCl_2_·2H_2_O, purity ≥99.0%), sodium carbonate anhydrous (Na_2_CO_3_, purity ≥99.8%), and R6G were bought from Tianjin Sailboat Chemical Reagent Technology Co., Ltd. (Tianjin, China). Cadmium nitrate solution (8.9 × 10^−3^ mol·L^−1^) was purchased from Dafengrui Instrument Sailing Co., Ltd. (Beijing, China). All reagents were analytical grades, no further purification is required.

### 2.2. Sample Preparation

The CuO FM was produced by hydrothermal method; 0.34 g CuCl_2_·2H_2_O and 0.22 g Na_2_CO_3_ were put into a beaker with 50 mL of distilled water and churned vigorously to dissolve. During the reaction, the solution color changed from colorless transparent to blue flocculent precipitate. After that, the synthesized compound was shifted to 100 mL of Teflon-lined autoclave (Zhengzhou Brocade Instrument Equipment Co. Ltd., Zhengzhou, China), and temperature was maintained at 200 °C for 20 h. Finally, the reaction sample was centrifuged (Hunan Hexi Instrument Equipment Co., Ltd., Changsha, China), then washed several times with deionized water and ethanol, and vacuum oven dried to 60 °C for 6 h. Thus, the CuO FM was obtained.

One milligram of CuO FM was dispersed in a test tube. Afterwards, 10 mL of absolute ethanol was poured into the above dissolution under ultrasonic oscillation (Shenzhen Acme Technology Co., Ltd., Shenzhen, China) for 25 min. 5 μL of CuO FM was dropwise dispersed on the silicon wafer (Zhejiang Lijing Optoelectronics Technology Co., Ltd., Hangzhou, China) and dried in air. After that, silver NPs were sputtered on the silicon wafer (0.6 cm × 0.6 cm) by electron beam evaporation (Zhongke Science Instrument Co., Ltd., Shenyang, China) (5.0 × 10^−4^ Pa) in the cavity and annealed at 300 °C for 2 h. Finally, the Ag@CuO FM material was obtained.

### 2.3. Sample Description

The crystal structures of CuO and Ag@CuO FM samples were identified using X-ray diffraction (XRD) (Rigaku, D/Max-3B, Tokyo, Japan). The morphologies of the samples were analyzed by scanning electron microscope (SEM) (Quanta FEG 250, FEI Company, Hillsboro, OR, USA). Transmission electron microscopy (TEM) and high-resolution electron microscopy (HRTEM) were acquired on a JEOL JEM-2010 instrument (JEOL, Tokyo, Japan) with an accelerating voltage of 200 kV. The elemental composition and valence of various elements in the samples were analyzed by X-ray photoelectron spectroscopy (XPS) (Thermo fisher Scientific, Kα^+^, Waltham, MA, USA). Raman spectra were performed for recording SERS signals by Raman spectrometer (Andor, Belfast, UK) with a 532 nm laser excitation source, a spot size of 0.785 μm^2^ and a 50× objective lens.

### 2.4. SERS Measurement

First, rhodamine 6G was dissolved in purified water to obtain R6G solutions with different concentrations. By adding purified water to the cadmium nitrate solution, a 1.0 × 10^−3^–1.0 × 10^−7^ M solution was prepared as the probe molecule. After that, 5 μL of R6G solution (or cadmium nitrate solution) with different concentrations were dropwise dispersed on the above-mentioned silicon wafer (0.6 cm × 0.6 cm) substrate containing Ag@CuO FM and then naturally dried. The detection of SERS was carried out under a 50× objective lens and confocal Raman system with excitation wavelengths of 532 nm and 0.785 μm^2^, respectively. Raman grating with a 1200 L/mm, the spectral data acquisition 3 times, each acquisition time is 10 s.

## 3. Results and Discussion

### 3.1. Structures Characterization and Elementary Composition

**Figure 1:**
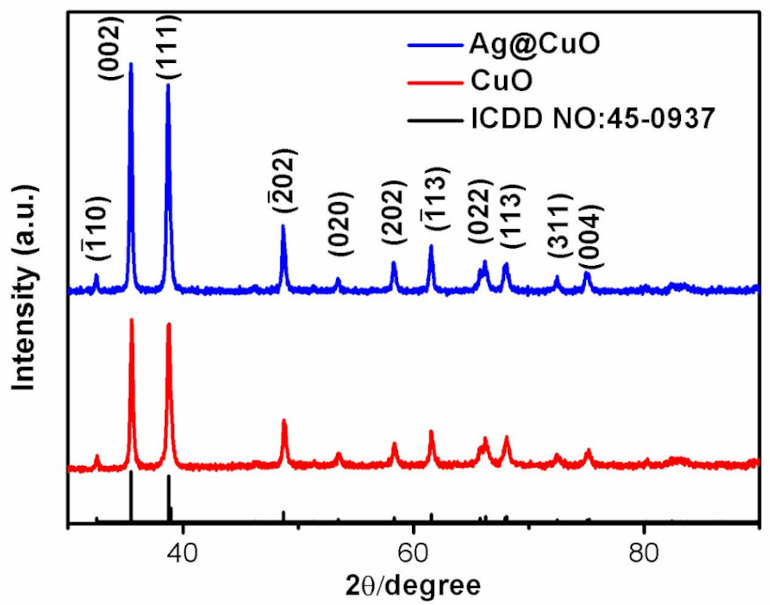
X-ray diffraction (XRD) pattern of CuO and Ag@CuO flower-like material (FM).

Figure 1 displays XRD patterns of CuO and Ag@CuO FM. All the diffraction peaks are indexed to the monoclinic structure of CuO (ICDD card No. 45-0937) with lattice parameters of *a* = 4.685 Å, *b* = 3.426 Å, *c* = 5.13 Å. The sharp diffraction peaks indicate good crystalline. The diffraction angles 2*θ* located at 32.49° 35.49°, 38.73°, 48.73°, 53.45°, 58.35°, 61.53°, 65.53°, 67.94°, 72.22°, and 75.02° correspond to the (1110), (002), (111), (202), (020), (202), (113), (002), (113), (311), and (004) crystal planes, respectively. All the blue diffraction peaks belong to the Ag@CuO FM. No Ag signals were detected since the thickness of the sputtered Ag NPs film is only 3 nm.

**Figure 2:**
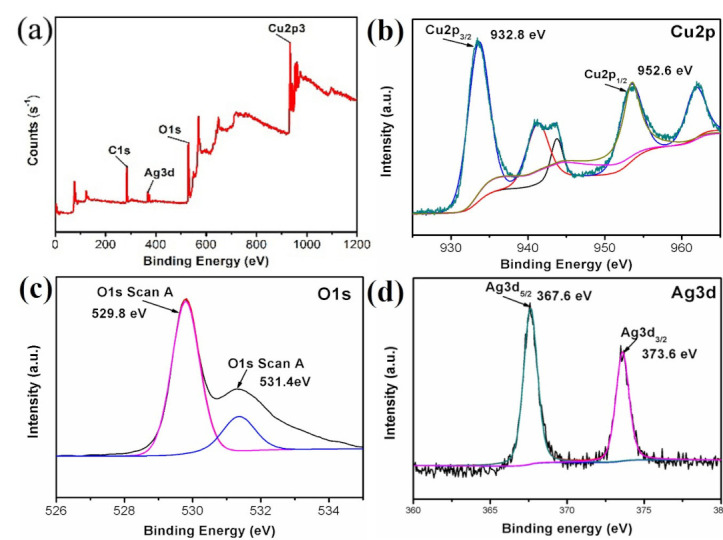
X-ray photoelectron spectroscopy (XPS) analysis of prepared Ag@CuO FM: (**a**) survey scan; (**b**) Cu 2p; (**c**) O 1s; (**d**) Ag 3d.

Figure 2a is the full-scan XPS spectrum of the Ag@CuO composite. It indicated that it is made up of Cu, O, and Ag elements. Figure 2b is the XPS bands for Cu 2p region. Two different peaks at 932.8 and 952.6 eV were attributed to Cu 2p_3/2_ and Cu 2p_1/2_, indicating the presence of Cu^2+^ ions, respectively [25]. There were two satellite peaks located at 961.2 and 942.8 eV in the spectrum [25,26]. Figure 2c shows the XPS band of O 1s. The characteristic peak of O 1s was composed of two peaks fitted by Gaussian (Avantage, version 5.984; XPS analysis software; Thermo Fisher Scientific: Waltham, MA, USA, 1995), of which one was located at 529.8 eV, which refers to the O-Cu bond energy, and the other was at 531.4 eV, corresponding to O_2_ adsorbed on the surface. XPS band of Ag 3d is shown in Figure 2d. The two obvious peaks of 367.6 and 373.6 eV represent Ag 3d_5/2_ and Ag 3d_3/2_, respectively, indicating that Ag was present in the sample [27]. The XPS results prove that the deposition of silver on the CuO FM surface is successful.

**Figure 3:**
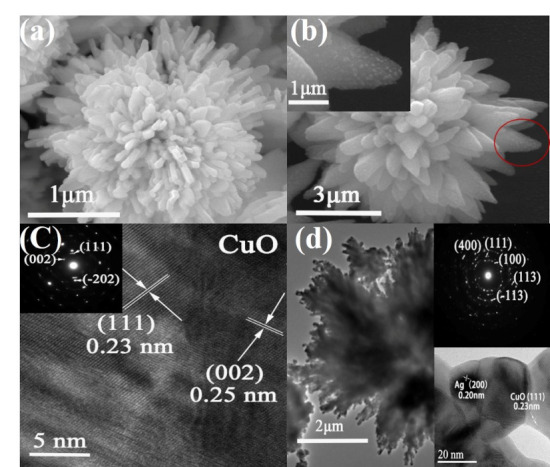
Scanning electron microscope (SEM), selected-area electron diffraction (SAED), and high-resolution electron microscopy (HRTEM) images of CuO FM and Ag@CuO FM. (**a**) CuO FM; (**b**) Ag@CuO FM; (**c**) CuO FM; (**d**) Ag@CuO FM.

Figure 3a,b displays the SEM image of CuO and Ag@CuO FM. It shows that the obtained CuO sample had a flower-like morphology, which was made of many plate-like building blocks. Ag NPs with average diameter of 20 nm were distributed on the surface of CuO FM, as presented in Figure 3b and its inset, further illustrating Ag NPs loaded on the surface of CuO FM.

The microstructure of Ag@CuO FM was further analyzed by TEM. TEM images of CuO and Ag@CuO FM are displayed in Figure 3c,d. Figure 3c is the HRTEM image of the CuO sample. The lattice spacing between nearby planes was 0.23 and 0.25 nm, respectively, corresponding to the (111) and (200) plane of CuO.

The inset of Figure 3c displays selected-area electron diffraction (SAED) of CuO FM. The diffraction rings were assigned to the (111), (112), and (002) crystal planes, indicating that CuO was polycrystalline. Figure 3d shows the HRTEM image of Ag@CuO FM. It is seen that Ag NPs and CuO FM had high crystalline and clear lattice stripes. The lattice spacing between nearby planes was 0.20 nm, which corresponded to the (200) crystal plane of the cubic Ag, while that of 0.23 nm was commensurate with the (111) crystal plane of CuO. The upper inset of Figure 3d shows the SAED image of Ag@CuO FM, which was indexed to the (111), (100), (113), (113), and (400) crystal planes, indicating the formation of Ag@CuO FM.

### 3.2. SERS Performance

#### 3.2.1. SERS Determination of R6G

**Figure 4:**
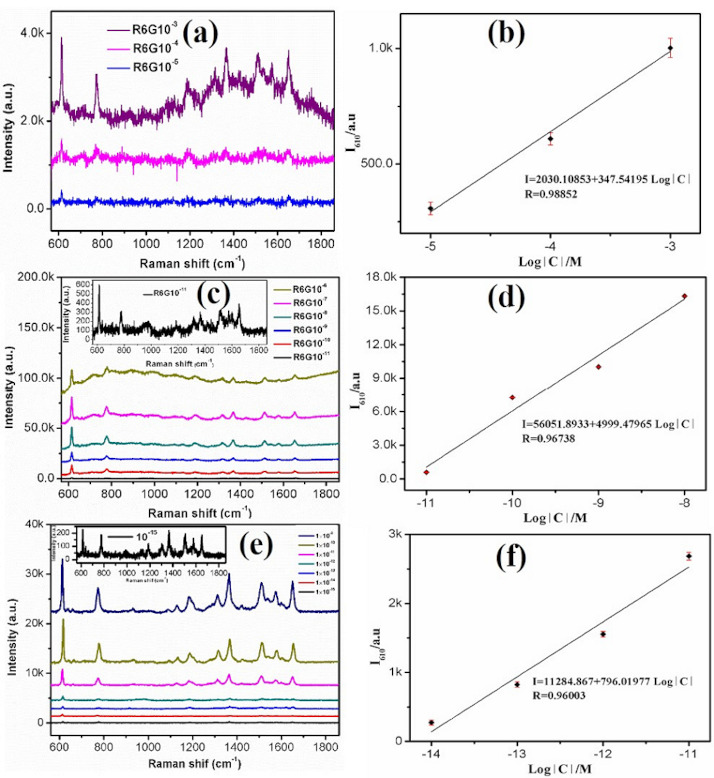
Surface enhanced Raman scattering (SERS) spectra of rhodamine 6G (R6G) with different concentrations on the different substrates: (**a**,**b**) 1.0 × 10^−3^–1.0 × 10^−5^ M on the CuO FM substrate; (**c**,**d**) 1.0 × 10^−6^–1.0 × 10^−11^ M on the Ag nanoparticles (NPs); (**e**,**f**) 1.0 × 10^−9^–1.0 × 10^−15^ M on the Ag@CuO FM substrate.

In order to assess the SERS property of CuO FM and Ag@CuO FM substrates, R6G was selected as a probe molecule. Figure 4 explains the SERS spectrum of R6G solutions with dissimilar consistencies on CuO FM substrate (Figure 4a), Ag NPs film with thickness of 3 nm (Figure 4c), and Ag@CuO FM (Figure 4e). The Raman strength of R6G on the CuO FM was much lower than those of the other two substrates. The calibration curves of average peak intensities at 610 cm^−1^ against the logarithmic consistence of rhodamine 6G for CuO FM, Ag NPs, and Ag@CuO FM substrates are envisaged in Figure 4b,d,f. Their corresponding linear relationships were achieved in the range of 1.0 × 10^−3^–1.0 × 10^−5^ M with *I* = 2030.1 + 347.54 log[*C*] (R^2^ = 0.988), 1.0 × 10^–8^–1.0 × 10^−11^ M with *I* = 56,051.8933 + 4999.4 log[*C*] (R^2^ = 0.967), and 1.0 × 10^−12^–1.0 × 10^−15^ M with *I* = 11,284.867 + 796.01977 log[*C*] (R^2^ = 0.96). Based on the definition of LOD, their corresponding LODs were calculated to be 3.86 × 10^−6^, 6.5 × 10^−12^, and 3.58 × 10^−16^ M, respectively, which approaches the single molecule level. Jayram et al. [17] reported superhydrophobic Ag modified copper oxide nanostructured for SERS research with a detection limit of 1.0 × 10^−10^ M for R6G, which was six orders of magnitude higher than our work. The representative peaks of R6G molecules were visible at the concentration in Figure 4. The peaks at 610, 774, and 1184 cm^−1^ were derived from the in-plane bended mode of the C-C-C rung, the out-of-plane bended mode of C-H, and the in-plane bended mode of C-H [18], respectively. Other peaks at 1364, 1511, and 1651 cm^−1^ contributed to the aromatic C-C stretched modes [18].

To effectively evaluate the Raman enhancement intensity of the substrate [28], the characteristic peaks 610 cm^−1^ of the probe molecule R6G were selected to calculate the enhancement factor (EF). The EF at 610 cm^−1^ was 3.99 × 10^10^. In comparison with other kinds of materials, we listed their EFs [15,16,17,18,19,29] in Table 1. It was found that the EF of our proposed substrate was the highest, indicating that the Ag@CuO FM substrate achieved excellent SERS performance.

**Figure 5:**
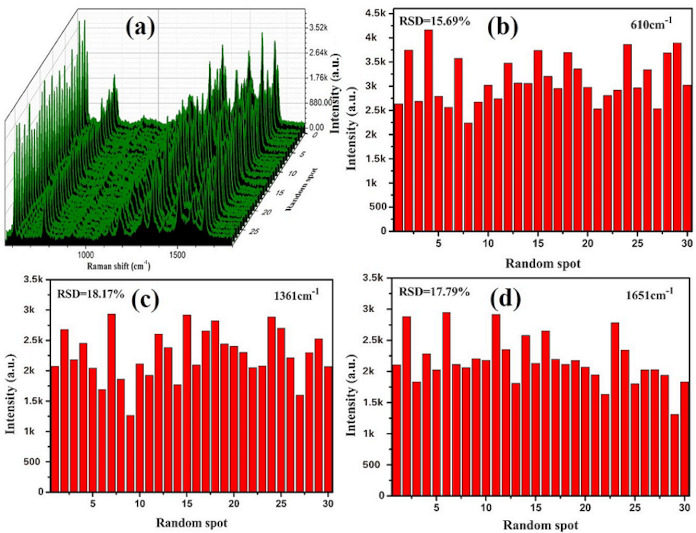
3D Raman spectra and relative standard deviation (RSD) values of the selected peaks: (**a**) 3D Raman spectra from 30 random spots; (**b**–**d**) RSD values are at 610, 1361, and 1651 cm^−1^ respectively.

To test the reproducibility and stability of prepared substrate SERS, Raman measurements were conducted in 30 spots by randomly moving the sample stage on the same substrate. A 1.0 × 10^−11^ M of R6G solution was utilized for measurements, as given in Figure 5. The signal strength of each characteristic peak of R6G was relatively consistent. The relative standard deviations (RSDs) of characteristic peaks at 610, 1361, and 1651 cm^−1^ were calculated to be 15.69%, 18.17%, and 17.79%, respectively.

#### 3.2.2. SERS Determination of Cd^2+^ Ions

**Figure 6:**
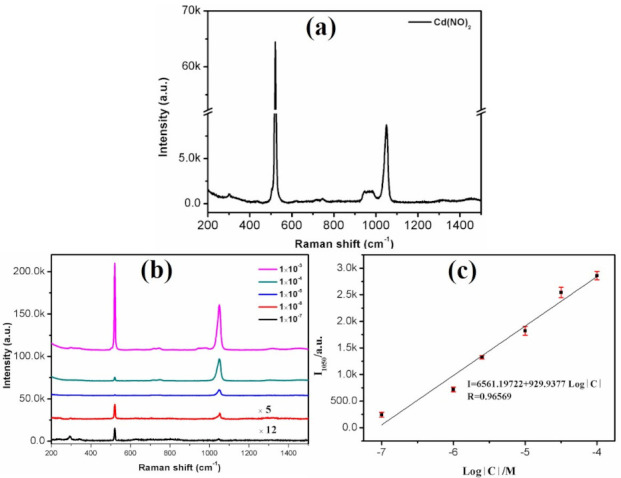
SERS spectra of cadmium nitrate at different concentrations. (**a**) cadmium nitrate solution; (**b**) at concentrations of 1.0 × 10^−3^–1.0 × 10^−7^ M on the Ag@CuO FM substrates; (**c**) linear calibration plot between SERS intensity and concentration.

In order to extend its practice application in the field of environmental monitor, we measured SERS spectra of Cd(NO_3_)_2_ solutions with different concentrations on the Ag@CuO FM substrates, as shown in Figure 6. It can be seen that the Ag@CuO FM substrate had a good enhancement effect. The measured minimum concentration was 1.0 × 10^−7^ M. The SERS spectrum of cadmium nitrate is shown in Figure 6a. The locations of the peaks were basically the same as those of its counterpart, as depicted in Figure 6b. The peak at 520 cm^−1^ originated from silicon. The peak at 1050 cm^−1^ was N-O symmetrical stretched mode, but to some extent its perturbation depends on the cationic ions of Cd^2+^ [32]. The peak at 300 cm^−1^ was the Cd-O tensile vibration mode [33]. The spectral characteristics of NO_3_^−^ at 715 cm^−1^ (*ν*_4_ mode) are usually used as evidence of ion-pair formation. The fitting curves of peak intensities at 1050 cm^−1^ and logarithmic density of the Cd(NO_3_)_2_ solution concentration is shown in Figure 6c.

We obtained a good linear relationship of *I* = 6561.19722 + 929.9377 Log[*C*] (R^2^ = 0.96569) in the range of 1.0 × 10^−4^–1.0 × 10^−7^ M. Similarly, LOD and EF were calculated to be 2.6 × 10^−8^ M and 6.9 × 10^3^, which were lower than the WHO and EPA recommended levels for safe drinking water. Its LOD was smaller than 3.87 × 10^−7^ M fluorescence method [3]. Compared with many reported SERS-based methods [1,30,31], we also inserted them in Table 1. The SERS properties of our proposed substrate were superior to those of the above-mentioned literatures [1,30,31] either for R6G or Cd^2+^ ions. In addition, we used this substrate to detect Pb^2+^ and Hg^2+^ by SERS and found that the detection concentration of Pb^2+^ was only up to 1.0 × 10^−2^ M (Figure S2 in Supporting Information), but for Hg^2+^ ions, no Raman signals were detected, revealing that our substrate had good selectivity to Cd^2+^ ions.

### 3.3. Mechanism

In order to investigate its mechanism, we measured SERS spectra of R6G on the bare silicon and CuO substrates. The results are below. As can be seen from Figure S1 (in Supporting Information), the minimum detection concentration (MDC) was 1.0 × 10^−4^ M when R6G was directly dropped on the silicon wafer for detection. Moreover, Raman signals were obtained only at a few points during measurements. Its MDC was lower than that of the CuO substrate. This result revealed that the SERS effect of the silicon substrate was not obvious, and the influence of silicon on the SERS detection was not significant. Based on that, we discussed its mechanism. The chemical enhancement mechanism (CM) and electromagnetic enhancement mechanism (EM) have been widely used for SERS enhancement. EM belongs to the physical model. The details are that it will cause local plasmon resonance on the surface, generating an amplified local electric field, and the SERS intensity of the molecule will naturally increase [34] when the incident ray is irradiated on the surface of the rough substrate. In this system, the Raman signal of the Ag@CuO FM substrate is enhanced due to the following synergies. First, the flower-shaped CuO FM provides a larger attachment area for Ag NPs, resulting in that more probe molecules absorbed on substratum and SERS performance are enhanced. Second, the CuO FM and Ag NPs combine to form a substrate with rough surfaces. This structure causes the incident photons generated by the excitation light to be amplified manyfold for SERS intensity. Third, electron transfers from Ag NPs to CuO FM occur when the incident laser is irradiated on the substrate, i.e., Ag NPs have a positive charge and CuO FM have a negative charge. Thus, coupling fields between Ag NPs and CuO FM are formed. In addition, the charge transfer between CuO FM and probe molecules also promotes higher SERS enhancement.

## 4. Conclusions

In conclusion, we prepared CuO FM by hydrothermal method and sputtered Ag nanoparticles on their surface to obtain Ag@CuO FM. Based on Ag@CuO FM as a substrate, SERS was used to detect carcinogens R6G and Cd^2+^, and the corresponding LOD could reach 3.58 × 10^−16^ and 2.6 × 10^−8^ M, respectively. The LOD of our obtained Ag@CuO FM for Cd^2+^ ions is lower than that of WHO recommended levels for safe drinking water and its sensitivity is lower than that examined by fluorescence method. This experiment opens up new possibilities for more sensitive, simple, and reliable exploration of Cd^2+^ ions in the environment.

## Figures and Tables

**Table 1:** Comparison of SERS EFs of our substrates and other substrates.

SERS Substrate	Molecule	LOD(M)	EF	Reference
Ag@ZnO	R6G	/	4.2 × 10^7^	[15]
Ag	R6G	/	/	[29]
Ag@CuO	R6G	1.0 × 10^−14^	3.3 × 10^9^	[18]
Ag-CuO	R6G	1.0 × 10^−9^	9.46 × 10^7^	[19]
Cu_2_O/Ag	R6G	1.13 × 10^−13^	2.7 × 10^9^	[16]
Ag@CuO	R6G	1.0 × 10^−10^	2.0 × 10^7^	[17]
Au	Cd^2+^	/	/	[30]
Au-TMT	Cd^2+^	2.9 × 10^−6^	/	[1]
SiO_2_@Au	Cd^2+^	/	/	[31]
Ag@CuO	R6G	3.58 × 10^−16^	3.99 × 10^10^	This work
Ag@CuO	Cd^2+^	3.87 × 10^−7^	6.9 × 10^3^	This work

EF—enhancement factor; LOD—limit of detection; TMT—trithiocyanuric acid.

## Supplementary Materials

The following are available online at https://www.mdpi.com/2079-4991/10/9/1664/s1, Figure S1: SERS spectrum of silicon substrate, Figure S2: SERS detection image of heavy metals Hg^2+^ and Pb^2+^ of Ag@CuO substrate.
