# Supplementary material for: Trace Cd2+ Ions Detection on the Flower-Like Ag@CuO Substrate
Source: Nanomaterials (Basel). 2020 Aug 25;10(9):1664. doi: 10.3390/nano10091664 (PMC7558543; doi:10.3390/nano10091664)
Supplement: Supplementary file 1 [file nanomaterials-10-01664-s001.pdf]

nanomaterials

Supplementary Materials:

Trace Cd^{2+} Ions Detection on the Flower-Like Ag@CuO Substrate

Mingming Cheng [1,2], Chenyan Li [1,2], Weijun Li [1,2] and Yingkai Liu [1,2,*]

1 Yunnan Key Laboratory of Opto-electronic Information Technology, Yunnan Normal University, Kunming 650500, China; Chengminng0526@126.com (M.C.); Lichenyan12@126.com (C.L.); Liweijun0009@126.com (W.L.)
2 Institute of Physics and Electronic Information, Yunnan Normal University, Kunming 650500, China
* Correspondence: ykliu@ynnu.edu.cn; Tel.: +86-871-6594-1166

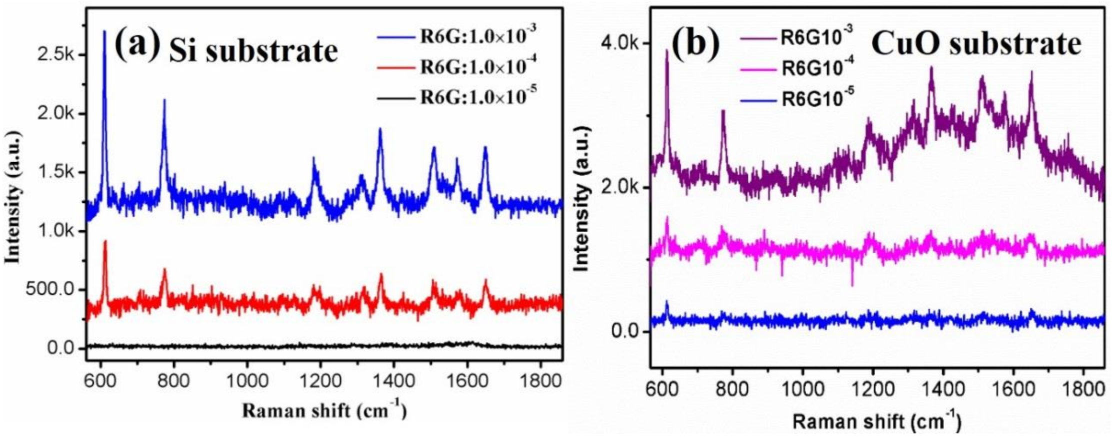

Figure S1. Surface enhanced Raman scattering (SERS) spectra of rhodamine 6G (R6G) with different concentrations on the different substrates. (**a**) 1.0×10^{-3} –1.0×10^{-5} M on the CuO flower-like material (FM) substrate; (**b**) 1.0×10^{-3}–1.0×10^{-5} M on the silicon substrate.

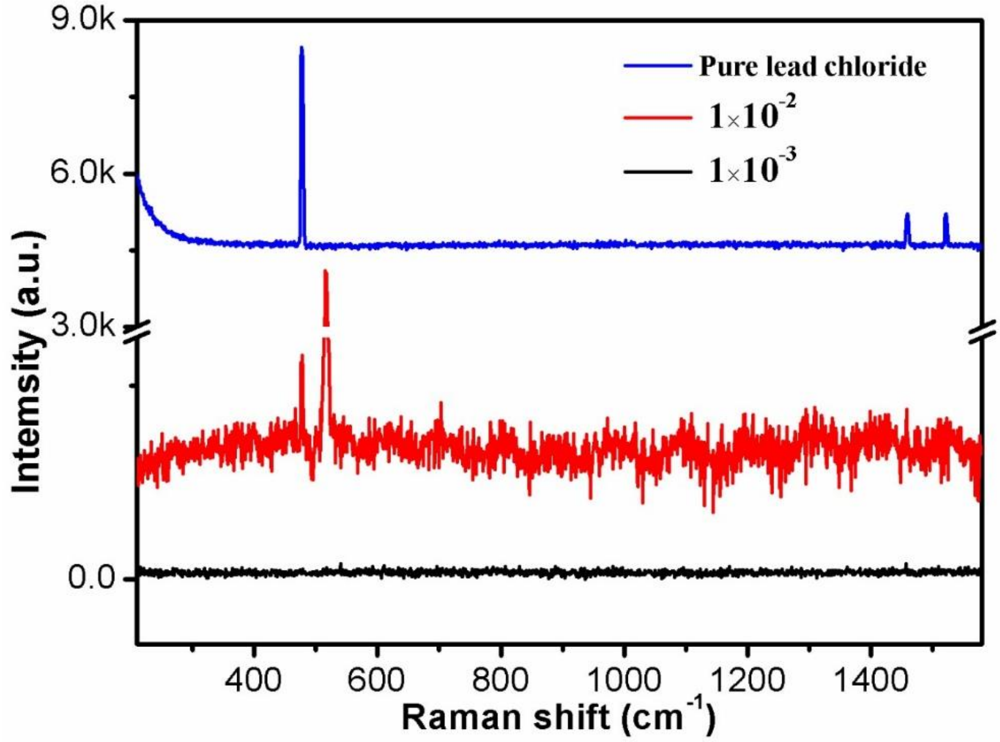

Figure S2. SERS spectra of lead chloride at different concentrations.
